# Gustation in insects: taste qualities and types of evidence used to show taste function of specific body parts

**DOI:** 10.1093/jisesa/iead018

**Published:** 2023-04-04

**Authors:** B H King, Panchalie B Gunathunga

**Affiliations:** Department of Biological Sciences, Northern Illinois University, DeKalb, IL 60115, USA; Department of Biological Sciences, Northern Illinois University, DeKalb, IL 60115, USA

**Keywords:** insect, taste, gustation, gustatory receptor, sensilla

## Abstract

The insect equivalent of taste buds are gustatory sensilla, which have been found on mouthparts, pharynxes, antennae, legs, wings, and ovipositors. Most gustatory sensilla are uniporous, but not all apparently uniporous sensilla are gustatory. Among sensilla containing more than one neuron, a tubular body on one dendrite is also indicative of a taste sensillum, with the tubular body adding tactile function. But not all taste sensilla are also tactile. Additional morphological criteria are often used to recognize if a sensillum is gustatory. Further confirmation of such criteria by electrophysiological or behavioral evidence is needed. The five canonical taste qualities to which insects respond are sweet, bitter, sour, salty, and umami. But not all tastants that insects respond to easily fit in these taste qualities. Categories of insect tastants can be based not only on human taste perception, but also on whether the response is deterrent or appetitive and on chemical structure. Other compounds that at least some insects taste include, but are not limited to: water, fatty acids, metals, carbonation, RNA, ATP, pungent tastes as in horseradish, bacterial lipopolysaccharides, and contact pheromones. We propose that, for insects, taste be defined not only as a response to nonvolatiles but also be restricted to responses that are, or are thought to be, mediated by a sensillum. This restriction is useful because some of the receptor proteins in gustatory sensilla are also found elsewhere.

## Introduction

An insect’s tough waxy cuticle provides support as well as protection from dehydration and from abiotic and biotic threats. However, this cuticle can interfere with detecting sensory cues that are necessary to survive and reproduce. Thus, transcuticular sense organs, sensilla, have evolved in response. Here we focus on gustatory sensilla, their basic structure, what they respond to, and how taste function is determined. Gustatory sensilla are the rough equivalent of taste buds (Thorne et al. 2004), and they are located on multiple parts of the insect body (King and Gunathunga 2023). This includes not only mouthparts, but also sometimes antennae, legs, wings, and ovipositors. Once a gustatory sensillum comes in contact with a potential resource, compounds may bind to taste receptor proteins, which are on taste dendrites within the sensillum. This may lead to acceptance or rejection of food (Chen et al. 2021, Oh et al. 2021, Piñero et al. 2021), oviposition sites (Ryuda et al. 2013, Sollai and Crnjar 2019, Dweck et al. 2021), or potential mates (Siva-Jothy and Stutt 2003, Warwick et al. 2009, Wada-Katsumata et al. 2018). Taste receptors on insect wings and other body parts may detect toxic compounds or infectious agents, thereby triggering grooming and thus removal of those compounds and agents (Yanagawa et al. 2019, Masagué et al. 2020).

Information on insect taste helps us understand how insects perceive their environment and may also facilitate new or improved pest control (van Naters and Carlson 2006). Tastants can be used to increase a pest’s consumption of insecticide or decrease a pest’s consumption of, e.g., crops, livestock, and wood in buildings. Understanding taste in beneficial insects, such as pollinators and predators of pests, may facilitate the development of toxic pest controls that are tasteful to the pest but distasteful to the beneficials.

The present review looks broadly across the literature on taste sensilla and what types of compounds they respond to, examining a broad range of insects, focusing on

1) what gustation is
2) taste qualities: the five canonical ones, whether there are more, and what the terminology means when talking about gustation in insects
3) appetitive versus aversive classification of tastes
4) basic morphology and neuroanatomy of insect taste organs (sensilla)
5) taste receptors on neurons in sensilla
6) the location of ‘taste receptor molecules’ and the definition of taste
7) types of evidence that indicate gustation

## What Gustation Is

The olfactory system and the gustatory system are both part of the chemosensory system. Olfaction is detection of volatile chemicals, i.e., as they move through air. This can include a response to CO_2_ or O_2_ moving through air (e.g., Jones et al. 2007, Agnihotri et al. 2016). Gustation is detection of nonvolatile chemicals, either liquids or solids. Gustation is sometimes called contact chemoreception (Städler 1984, Chapman 2003), although detection at a close range is sometimes more accurate. Certain taste sensilla in the mouthparts of caterpillars of *Manduca sexta* (Linnaeus) (Lepidoptera: Sphingidae) respond at less than ~0.6 mm (Dethier 1972, Städler and Hanson 1975). When presented at 2–3 mm, the legs of desert locusts, *Schistocerca gregaria* (Forskål) (Orthoptera: Acrididae), respond to some, although not all, tastants (Newland 1998). When partially volatile compounds, e.g., DEET (*N*, *N*-diethyl-m-toluamide), ammonia, carbonation, water, and polyamines, as well as certain acids, pheromones, and fatty acids, are encountered, olfactory organs may respond to the volatized part and gustatory organs to the nonvolatized part (Delventhal et al. 2017, Montell 2021).

The body part(s) with taste function are not the same across all insect taxa (reviewed in King and Gunathunga 2023). A given body part may have both taste and olfaction functions, e.g., labellum of adult *Anopheles gambiae* Giles (Diptera: Culicidae) (Kwon et al. 2006) and maxillary galeae (proboscis) of adult *M. sexta* (Reiter et al. 2015, Haverkamp et al. 2016). Alternatively, a given body part may not have both taste and olfaction. For example, gustation, but not olfaction, appears to be a function of wings of adult *Drosophila melanogaster* Meigen (Diptera: Drosophilidae) (Agnel et al. 2017). For aquatic organisms, even though sensed chemicals are not airborne, researchers still talk about taste versus olfaction, but ‘olfaction’ is based on similarity to the olfactory organs of terrestrial organisms in terms of sensilla location, structure, or response (Crespo 2011, Rebora et al. 2019).

The presence of ‘gustatory’ or ‘odor’ in a term does not always indicate that only taste or smell, respectively, is involved. The terms sometimes simply reflect the function that was first discovered or hypothesized. For example, some odorant binding proteins are now known to be involved in taste (Sun et al. 2017a, 2017b, Rihani et al. 2021). Similarly, some proteins in the gustatory receptor family (GRs), which are products of *Gr* genes, are now known to be involved in olfaction (Jones et al. 2007, Kwon et al. 2007). The GR in GRN (gustatory receptor neuron) can also be confusing because one might expect it to express GR protein, but it can simply mean a nerve cell thought to be involved in taste (e.g., Chen and Dahanukar 2017).

## Taste Qualities

Each compound that an insect tastes, i.e., each tastant, may elicit a unique spatiotemporal neural representation (Reiter et al. 2015). Tastants often vary in the electrophysiological response that they generate, in the latency of response, as well as in the pattern of the action potential spikes, the spikes’ height, shape, and frequency (e.g., Weiss et al. 2011). However, for convenience, tastants have been grouped into taste qualities, initially, sweet, bitter, sour, and salty (Minnich 1921), with umami (savory) later added (Liman et al. 2014). These five are referred to as the canonical taste qualities (taste modalities). Insects respond to all five; however, it is important to remember that compounds within a taste quality may cause different perceptions in insects than in humans. In addition, how a particular taste quality is defined for insects is not consistent, e.g., a compound may be described as bitter based on i) human perception of the compound as bitter, ii) an insect-exhibiting an aversive response, or iii) the compound activating a receptor that responds to other compounds that are considered bitter (Reiter et al. 2015). Insects show both similarities and differences in their responses to various tastants compared to humans. For example, honey bees respond positively to a subset of the compounds that are sweet to humans (Bestea et al. 2021).

Sweet is especially associated with sugars (e.g., Agnihotri et al. 2016, Bestea et al. 2021) but also with sugar alcohols, which are important for some Lepidoptera (Agnihotri et al. 2016, Kikuta et al. 2016, Xu 2020) and other insects (LeDue et al. 2015, Takada et al. 2017). Insects also respond to some artificial sweeteners that are not sugars or sugar alcohols, e.g., Acesulfame K (e.g., King et al. 2019). Bitter is associated with compounds like caffeine, denatonium, and quinine, compounds that may be toxic and/or deterrent (French et al. 2015, Sollai et al. 2015, Ntie-Kang 2019, Muñoz et al. 2020). Bitter compounds are extremely diverse in chemical structure (Meyerhof et al. 2011). Sour is associated with certain acids, like acetic acid, citric acid, hydrochloric acid, and lactic acid (Crnjar et al. 1989, Masala et al. 2014, Mi et al. 2021, Stanley et al. 2021). Salty is associated with sodium and other mineral ions, usually NaCl, but also KCl and others (Seada et al. 2018, Masagué et al. 2020, McDowell et al. 2022). Umami (savory) is associated with certain amino acids (Schoonhoven et al. 2005, Zhang et al. 2011, Croset et al. 2016, Bestea et al. 2021, Aryal et al. 2022).

Additional taste qualities are being considered (Keast and Costanzo 2015, Besnard et al. 2016), including for insects (Chen and Dahanukar 2020). For insects this includes: water (hypoosmolarity) (Cameron 2010, Solari et al. 2010, Popescu et al. 2013); fatty acids; and carbonation = soluble CO_2_, which is a fermentation product (Fischler et al. 2007, Sanchez-Alcaniz et al. 2018). Other compounds that some insects can taste but which may not easily fit into current taste qualities include: RNA and ribonucleosides, which are essential macronutrients for *D. melanogaster* larvae (Mishra et al. 2018); polyamines such as putrescine and cadaverine (Hussain et al. 2016b, Aryal and Lee 2021); ammonia (Delventhal et al. 2017); reactive electrophiles, such as N-methyl maleimide and allyl isothiocyanate, the latter being the source of the pungent taste of mustards such as wasabi and horseradish (Kang et al. 2010); the divalent cation calcium (Ca^2+^) in CaCl_2_, which is found in some plants and reduces survival at high concentrations (Lee et al. 2018); zinc (Luo et al. 2022), as well as other metals (Luo et al. 2022, Xiao et al. 2022); and bacterial lipopolysaccharides (Chen and Dahanukar 2020 and references therein). Many but not all blood-feeding insects feed more when given ATP and/or ADP, which occur in blood (reviewed in Barrozo 2019). Some caterpillars have taste receptors that respond to ecdysteroids that occur in some plants and are insecticidal (Calas et al. 2006, Rharrabe et al. 2011 and references therein). Fermenting foods contain the monoamine histamine, which *D. melanogaster* taste and avoid at high concentrations (Aryal and Lee 2021).

Hydrogen peroxide (H_2_O_2_) has been suggested as a noncanonical taste quality that *D. melanogaster* responds to (Chen and Dahanukar 2020). However, humans perceive it as bitter (ATSDR 2002). In *D. melanogaster*, the response of bitter sensing neurons to H_2_O_2_ is an indirect way for females to avoid laying eggs in bright locations; H_2_O_2_ production is light-induced (Guntur et al. 2017).

Some insects respond to certain pheromones by taste, including compounds on the surface of conspecifics, such as long-chain cuticular hydrocarbons (CHCs) that have no or low volatility (Lacaille et al. 2007, Starostina et al. 2009, 2012, Ozaki and Wada-Katsumata 2010), although some such CHCs can also be detected at short distances by the olfactory system (Brandstaetter et al. 2008). Pheromones that insects taste are called contact pheromones. Some contact pheromones are aphrodisiac or antiaphrodisiac (Fan et al. 2013, Depetris-Chauvin et al. 2015, Ahmed et al. 2019). Males sometimes court other males in some insects (Dukas 2010, Benelli and Canale 2012), and in many populations of *D. melanogaster*, a male is inhibited from doing so when his labial palps and legs contact the CHC 7-tricosene on another male (Lacaille et al. 2007). The 7-tricosene stimulates a taste receptor that also responds to bitter compounds.

The categorization of tastants for insects is further complicated because some are based on human taste perception and some on chemical structure, yet not all compounds within a chemical structural group have the same general taste to humans (Bohm 1998). For example, to humans, some amino acids provide umami, but other amino acids taste sweet, bitter, tasteless, or bittersweet (Jakinovich 1981). To humans, the flavonoid naringin, which is in grapefruit, tastes bitter; whereas the flavonoid neoastilbin tastes sweet, even though its stereoisomer astilbin does not. Stereochemistry also sometimes affects taste response of insects (Roessingh et al. 1999, Hughes et al. 2015, Sparks and Dickens 2016).

For humans, some ‘sweeteners’ also create additional sensations. Ace-K and sodium cyclamate are described as sweet but also as metallic, astringent, or cooling (Świa̧der et al. 2009). Perhaps insects also detect these aspects. In some insects, tastants such as capsaicin (in hot peppers), allyl isothiocyanate (in wasabi), and menthol (in some mints) activate temperature receptors, receptors that typically respond to hot or cold (Maliszewska et al. 2018). In American cockroaches, *Periplaneta americana* (Linnaeus) (Blattodea: Blattidae), such compounds can even change body temperature and thermoregulatory behavior.

Insects, sometimes but not always, distinguish among tastants within a taste quality. For example, *M. sexta* caterpillars differentiate the sugars sucrose and trehalose (Glendinning et al. 2006). They also differentiate between the bitter compounds salicin and aristolochic acid, but not those from caffeine. *Heliothis virescens* (Fabricius) (Lepidoptera: Noctuidae) moths differentiate bitter compounds like quinine and sinigrin (Jorgensen et al. 2007). Kissing bugs can differentiate quinine and caffeine (Asparch et al. 2016). However, kissing bugs and honey bees do not differentiate the salts NaCl and KCl (Pontes et al. 2017, Guiraud et al. 2018, Masagué et al. 2020). *D. melanogaster* do not differentiate fructose and glucose, or the bitter compounds berberine, caffeine, quinine, and denatonium benzoate (Masek and Scott 2010). Not all insects or all body parts respond to all taste qualities, e.g., in honey bees, water does not stimulate the part of the maxilla tested by Whitehead and Larsen (1976a).

## Appetitive or Aversive

Insect neurons, tastants, and responses to tastants are sometimes classified as appetitive, phagostimulatory, or attractant versus as aversive or deterrent (e.g., Tsuneto et al. 2019, Zhou et al. 2021). In feeding, appetitive behavior includes biting or a proboscis extension response (PER) and ingestion. PER is the extension of retracted or folded up mouthparts in apparent preparation for contacting food and feeding, often in response to stimulation of a body part other than the part of the mouth that will do the feeding, e.g., stimulation of the antennae or tarsi or labial palps. PER has been used to test taste in various flies (King et al. 2019, Piñero et al. 2021), lepidopterans (Hostachy et al. 2019, Liu et al. 2020), bees (Ruedenauer et al. 2019, Ali et al. 2021), ants (Guerrieri and d’Ettorre 2010), and kissing bugs (Páez-Rondón et al. 2018). Aversive behavior includes avoidance, and inhibition of PER or of biting.

Compounds that humans consider appetitive, such as sweet compounds, are often, but not always, appetitive to insects. Similarly, compounds that humans generally consider aversive, such as bitter compounds, also tend to be aversive to insects (Scott 2018). Among compounds that are sweet to humans, an insect’s ranking from most to least appetitive can differ from the pattern for humans (King et al. 2019). For example, sucralose, Acesulfame potassium, and sodium cyclamate are much less likely than sucrose to elicit PER by house flies, yet humans report them as being much, much sweeter than sucrose (King et al. 2019). In addition, ranking may depend on the response being measured, e.g., PER versus consumption, and may depend on whether the compound is solid or in solution (King et al. 2019, Choi et al. 2020).

Whether a given compound is appetitive or aversive can vary even among closely related species (e.g., Sun et al. 2021a) and within species (e.g., Devineni et al. 2019). Many bitter and/or aversive compounds that plants produce evolved as herbivore repellents. However, subsequently, some insect specialists evolved to feed on those plants, including evolving to use ‘repellents’ as a means to identify plants for consumption or oviposition (e.g., Hopkins et al. 2008). Within a species, whether a given compound is appetitive or aversive can vary depending on concentration. For example, *D. melanogaster* prefer mildly acidic or salty food yet avoid highly acidic or salty food (Jaeger et al. 2018, Mi et al. 2021). Within a species, nutritional needs influence the gustatory response (Blystone 2015), e.g., depending on: i) ovarian maturation stage, because egg production often creates a need for protein (e.g., Solari et al. 2015); ii) mating status (e.g., Hussain et al. 2016a), because courtship may use up energy or particular nutrients used to attract mates and protect offspring (e.g., Eisner et al. 1996), and iii) prior exposure to the compound or other tastants (e.g., Zhou et al. 2010, Asparch et al. 2016, Bestea et al. 2021). Much research has been on individual tastants, but gustatory response to a mixture may be more or less than the sum of the responses to the individual tastant components (e.g., Omand and Dethier 1969, Städler and Schöni 1991, van Loon et al. 2008, Glendinning 2015).

## Morphology and Neuroanatomy of Taste Sensilla

**Fig. 1.**
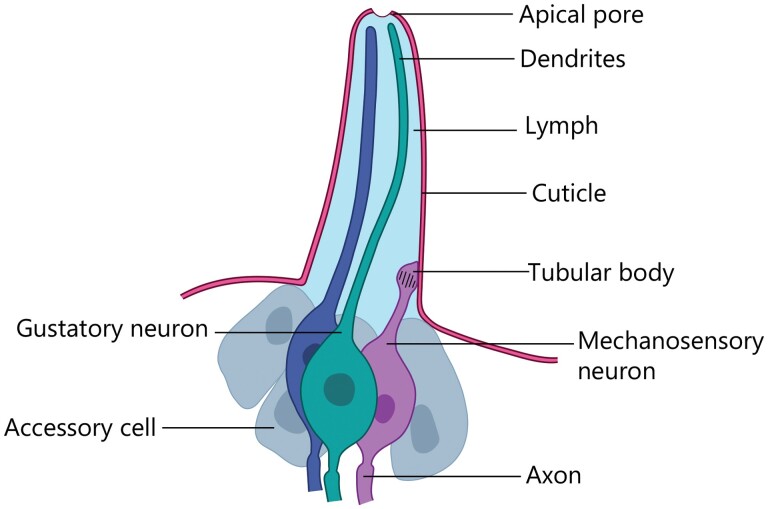
Simplified example of a gustatory sensillum, with two gustatory neurons (dark blue, green), one mechanosensory neuron (magenta), and three accessory cells (grey). Image by Nora Hunter CC BY SA.

Each taste sensillum contains one or more neurons, as well as some nonneural cells, also called accessory or support cells (e.g., trichogen, tormogen, and/or thecogen cells); and the dendrites are protected by extensions of the cuticle (Fig. 1) (e.g., Chun and Schoonhoven 1973, Mitchell et al. 1999, Shanbhag et al. 2001). Each neuron is bipolar, with its dendrite extending outward from the neural body into the cuticular shaft of the sensillum and the axon extending in the other direction and connecting to the insect’s central nervous system. A pore at the tip of the sensillum’s shaft allows tastants to reach the lymph that surrounds the dendrite(s). If the dendrite has the appropriate protein receptor(s) on it, the tastant activates the receptor(s), causing the neuron to produce a train of action potentials.

At first glance, many of the sensilla on an insect look like randomly scattered hairs of variable length and width. However, within a given sex and developmental stage of a species, the location of sensilla on a given body part is relatively stereotypical, as is the shape of a sensillum at a given location. This has been documented, for example, for the labellum of *Ae. aegypti* (Hill and Berry Smith 1999) and the tarsi and labellum *of D. melanogaster* (Ling et al. 2014, Freeman and Dahanukar 2015). But in some insect species, variation exists. In females of the moth *Spodoptera littoralis* (Boisduval) (Lepidoptera: Noctuidae) studied by Seada et al. (2018), the fifth tarsomere of the right foreleg has six of one type of taste sensilla in 90% of individuals and eight in the other 10%. In larvae of the Colorado potato beetle, *Leptinotarsa decemlineata* (Say) (Coleoptera: Chrysomelidae), studied by Messchendorp et al. (1998), the epipharynx has either 20 or 21 sensilla. In adult *D. melanogaster* studied by Shanbhag et al. (2001), the number of taste pegs on the inner surface of the labellum shows some variation even within each sex, and in those studied by Ling et al. (2014), about 10% of female flies differ from the most common pattern of tarsal sensilla in females.

Besides being named by function, sensilla are also named for features like their external cuticular shape, length, and width, and whether they are in a socket. Names include trichoidea, chaetica, basiconica, styloconica, coeloconica, and placoidea (Schneider 1964, Zacharuk and Shields 1991). Different terms have been used for the same shape of sensillum (Sevarika et al. 2021, Zhu et al. 2021). For example, in *D. melanogaster*, taste sensilla are often classified as bristles, pegs, and pores (Shanbhag et al. 2001, Scott 2018, Chen and Dahanukar 2020). However, taste pores are also called no-shaft terminal pore sensilla (Stocker 1994), pharyngeal hairless sensilla (Chen and Dahanukar 2020), or taste papillae (Shanbhag et al. 2001). In lepidopterans at least six different shapes of taste sensilla have been named, including various hair-like and peg-like ones, as well as thumb-shaped ones with a cone at the tip, which are called sensilla styloconica (e.g., Ma et al. 2019, Xu 2020).

Sensilla are also often grouped as aporous, uniporous, or multiporous, which roughly but imperfectly correspond to mechanosensory, or the less common hygro and/or thermosensilla; gustatory; and olfactory, respectively (Altner and Prillinger 1980, Steinbrecht 1984, Zacharuk and Shields 1991). Limitations of using uniporous to assign a taste function are discussed below under the heading, ‘Morphological and neuroanatomical evidence’.

The location of sensilla can provide an insect with spatial information about its environment. For example, in the blow fly *Phormia regina* (Meigen) (Diptera: Calliphoridae), the proboscis extends toward whichever labellar sensilla are stimulated by a tastant (Yetman and Pollack 1987). When a leg on just one side is stimulated with sucrose, the mosquito *Culiseta inornata* (Williston) (Diptera: Culicidae) turns to that side (Pappas and Larsen 1978). Even very similarly shaped taste sensilla near each other on the same body part may respond differently to different tastants, as is the case for the medial versus lateral sensilla styloconica on the maxillary galea of at least some caterpillars (Waladde et al. 1985, Chen et al. 2022, Tsuchihara et al. 2022, and references therein).

Some taste sensilla have a mechanosensory neuron in addition to gustatory receptor neuron(s) (GRN). For clarity, such sensilla may be described as having a ‘touch and taste function’ (Rebora et al. 2016a) or as being bimodal contact chemo-mechano sensilla (Krenn 1998, Shields 2009). However, even when a mechanosensory neuron is present, such sensilla are often referred to as just ‘gustatory sensilla’ or ‘taste sensilla’. ‘Contact chemosensilla’ indicates a taste function, with or without a mechanosensory function (e.g., Rist and Thum 2017). The ‘contact’ in ‘contact chemo-’ terms does not refer to mechanosensation, rather the ‘contact’ refers to taste as opposed to olfaction, because tastants generally have to be contacted because they are not volatile (Städler 1984, Montell 2021, Shields 2021).

Taste sensilla that do not also respond to touch are sometimes described as unimodal because they involve only one sensory ‘channel’ or ‘mode’, in this case only taste, not also mechanosensory, olfactory, auditory, or visual (Hallberg and Ahman 1987). In this context, referring to gustatory sensilla as ‘unisensory’ might be clearer because sometimes ‘unimodal’ instead means within a sensory channel, e.g., responding to only one taste quality (as in Maier et al. 2020).

The number of GRN reported for insect sensilla is 1–9 (Altner and Prillinger 1980). The range seen in adult *D. melanogaster* is 1–8, with most of the taste sensilla having 1–4 (e.g., Table 1 in Montell 2009). The sensilla in *D. melanogaster* larvae each have 1–4 GRN (Rist and Thum 2017). Gustatory sensilla of honey bees have 3–5 (Bestea et al. 2021). These numbers are common for other insects as well (e.g., Mitchell et al. 1999, Rebora et al. 2016b, Sevarika et al. 2020). The number of neurons within a taste sensillum tends to be consistent for a given sensillum morphology at a given body location, but some variations occur (Pappas and Larsen 1976). For example, on the labellum of adult *D. melanogaster*, short taste bristles typically have four chemosensory neurons, and intermediate taste bristles typically have two chemosensory neurons, but both short and intermediate bristles can have anywhere from two to four chemosensory neurons.

An insect may be able to detect a given taste quality with more than one body part. Even if detection is with a single body part, the taste sensilla that are activated may not all be the same shape and length and may not all have the same number of neurons (e.g., Table 2 in Montell 2021). Some sensilla are broadly tuned, i.e., respond to a wide range of tested tastants, and others seem to be narrowly-tuned (Weiss et al. 2011, Ling et al. 2014). A response of a sensillum to multiple tastants can result from the sensillum’s neurons differing in what tastants they are sensitive to, or from individual neurons responding to more than one tastant.

## Tastants Activate Receptors on Neurons in Sensilla

When there are multiple chemosensory neurons in a taste sensillum, not all of them respond to the same set of tastants. For example, in some Lepidoptera, some sensilla contain one GRN responding to salt, one or more to sugars, and one to bitter compounds (Mitchell et al. 1999, Seada et al. 2018). In adult *D. melanogaster*, some sensilla each contain one GRN responding to sugars, one to water, one to low concentrations of salt, and one to high concentrations of salt (Table 2 in Montell 2021). However, specifying, for example, the presence of a ‘sugar GRN’ (= sugar cell, = sweet neuron) does not indicate that the neuron is activated by all different sugars or to all sugars with equal intensity (e.g., Tooming et al. 2012, Parkinson et al. 2022). In addition, a sugar GRN may respond to compounds other than just sugar. For example, a particular sweet neuron in *D. melanogaster* responds to sugars, glycerol (which is sweet to humans), low Na+, fatty acids, and acetic acid. A particular bitter neuron in *D. melanogaster* responds not only to commonly tested bitter compounds, but also to acids, high concentrations of salts, microbial lipopolysaccharides, and cool temperatures (Freeman and Dahanukar 2015, Table 2 in Montell 2021). A single taste neuron that responds to both sugar(s) and amino acid(s), and for at least some species also glycoside(s), has been reported, e.g., blow flies (Steinbrecht 1984), the tiger moth caterpillar *Grammia geneura* (Strecker) (Lepidoptera: Arctiidae), the red turnip beetle, *Entomoscelis americana* Brown (Coleoptera: Chrysomelidae), and the adult Colorado potato beetle, *L. decemlineata* (Bernays et al. 2000 and references therein). Referring to GRN as a ‘sugar best cell’ or ‘salt-best cell’, etc. is a concise way to make clear that a GRN responds to other tastants as well and that it is being described by what it is best known for responding to and/or is particularly sensitive to (Schoonhoven et al. 2005, Shields 2021).

A given tastant may stimulate some taste neurons but also inhibit or synergize others (Dethier 1971, Xiao et al. 2022). For example, a bitter compound may activate bitter neurons while also reducing the sensitivity of sweet neurons and water neurons (French et al. 2015, Benton 2017, Seada et al. 2018, and references therein). Japanese carpenter ants, *Camponotus japonicus* Mayr (Hymenoptera: Formicidae), provide an example of taste synergism. The ants feed from a glucose-glycine secretion of a caterpillar that is an ant-nest parasite. The glycine synergizes the positive effect of glucose on the firing of a taste sensillum on the ant’s labial palps, increasing feeding by the ant (Wada et al. 2001).

The range of tastants that a given GRN responds to may be broad or narrow. When the GRNs in the proboscis of *M. sexta* moths were exposed to 12 tastants, including water, sugars, salts, and bitter compounds, some GRNs were very specific, responding to just one tastant, but about 60% of tested GRNs responded to more than one tastant, including tastants from different taste qualities (Reiter et al. 2015). Some GRNs responded to all 12 tastants. Similarly, when *D. melanogaster* larvae were tested with tastants from each of the five canonical taste modalities (sweet, bitter, sour, salt, amino acids), more than 30% of GRNs responded to more than one taste modality, i.e., were multimodal for taste (Maier et al. 2021). At least in *D. melanogaster* larvae, a single GRN may even be activated by tastants of presumed opposite effects, e.g., a sugar, which was presumed attractive, and bitter or high salt, which were presumed aversive (van Giesen et al. 2016, Maier et al. 2021).

Genes for insect taste receptors are found within at least five gene families: gustatory receptors (GRs), ionotropic receptors (IRs), pickpocket channels (PPKs), transient receptor potential channels (TRPs), and G protein-coupled receptors (GPCRs), specifically opsins (Montell 2021). Receptor proteins from one or more of these receptor families are found in a diversity of insects, e.g., from Diptera, Lepidoptera, Coleoptera, Hymenoptera, Hemiptera, Phthiraptera, Orthoptera, and Blattodea, although few species have been examined in each order (Xu et al. 2017b, Xu 2020, Latorre-Estivalis et al. 2021, Yin et al. 2021, Ortega Insaurralde et al. 2022, and references therein). In all five of these gene families, only some of the genes and proteins are involved in taste (Ni 2020, Montell 2021). Furthermore, a protein or gene that is very similar to one that is involved in taste in one species, e.g., *D. melanogaster,* may not itself have a taste function. This is because similar sequences can evolve to different functions in different species, and a single gene can affect more than one aspect of phenotype, even within a single individual, e.g., may lead to the production of different proteins in different body parts.

Based on sequence data, the GRs are classified into clades named: CO_2_, D-fructose, nonfructose sugar 1 and 2, and bitter and other (Sánchez-Gracia et al. 2009, Fig. 2. in Agnihotri et al. 2016). It is important to remember that being in a particular clade does not necessarily reflect function. For example, the bitter receptor family was so named because some of its receptors respond to bitter, but the function remains unknown for most of the more than 100 genes in the clade, and at least one responds to proline, an amino acid, although not to four other amino acids (Xu et al. 2016). Naturally occurring proline is perceived by humans as sweet-bitter (Schiffman et al. 1981, Birch and Kemp 1989, Kawai et al. 2012).

Which tastants a given neuron in a sensillum responds to depends on the neuron’s combination of specific receptor proteins. Even the response to a single tastant may involve multiple different taste receptor proteins. A single taste neuron often contains receptors from more than one receptor family, e.g., both GRs and IRs (Chen and Dahanukar 2017). Some receptors only work in the presence of another receptor (Isono and Morita 2010).

## The Location of ‘Taste Receptor Molecules’ and the Definition of Taste

Examples of tastants that activate each of the different taste receptor proteins in different body parts are available for *D. melanogaster* adults (Chen and Dahanukar 2020). A search for genes similar to those that encode these receptors has begun in other insect species (e.g., Sparks and Dickens 2017, Latorre-Estivalis and Lorenzo 2019, Xu 2020). A small subset of these studies have also assessed what body parts these receptors are expressed on and what tastants the receptors bind to. Such molecular studies have revealed taste receptors not only on various peripheral body parts and in the digestive tract (Park and Kwon 2011a, King and Gunathunga 2023), but also in some other internal body parts. Internal organs in which receptor proteins from the GR family are expressed include: the central nervous system (Thorne and Amrein 2008, Mishra et al. 2013, Fujii et al. 2015, Jung 2015, Mang et al. 2016a, 2016b), the fat body (Jung 2015, Koenig et al. 2015), male and female reproductive organs (Park and Kwon 2011b, Miyamoto et al. 2012, Miyamoto and Amrein 2014, Mang et al. 2016a), Malpighian tubules (Mang et al. 2016a), the silk gland of silkworms (Mang et al. 2016a), and the gland that makes royal jelly in honey bees (Jung 2015). At least some of these receptor proteins are involved in regulating nutrient levels, which may in turn affect feeding. Trehalose is the major hemolymph sugar of insects. When it binds to a GR in one of these internal organs, should this automatically be considered taste? To avoid the term ‘taste’ becoming so broadly defined as to be almost meaningless, we suggest that the term ‘taste’ be restricted to when the response is thought or known to begin at a sensillum. Evidence of sensilla in internal organs other than parts of the digestive tract is lacking. As with many terms, a clear distinction between ‘internal taste’ and other types of internal chemical signaling may not always exist.

## Evidence of Gustation

Types of evidence that reveal the involvement of taste include: electrophysiological, behavioral, morphological and neuroanatomical, and molecular. The molecular basis of taste has been well-reviewed elsewhere (e.g., Montell 2021), so here we focus on the other types of evidence. It is tempting to consider electrophysiological evidence as the strongest type, but electrophysiological recording is not always feasible, because the equipment is expensive and because of technical challenges, such as a sensillum being difficult to access as it is very small or surrounded by many other sensilla or embedded in lots of chitin (Alabi et al. 2014, Delventhal et al. 2014) Wings have been notably difficult to consistently record from (Yanagawa et al. 2014). A limitation of both electrophysiological and behavioral evidence is that a lack of response to tastants does not rule out a taste function because a response to tastant(s) that were not tested cannot be ruled out. With certain types of evidence, different responses to different tastants might be a result of the organism responding to differences among the tastants in odor, texture, or appearance, not just taste. Thus, nontaste cues need to be ruled out. A response to odor can be ruled out if the tastant is nonvolatile.

Electrophysiological and behavioral evidence of taste are based on tests of different tastants or concentrations. Such tests should avoid confounding effects of the order of presentation (Evans and Mellon 1962). Such effects can result if evaporation from solutions occurs across the duration of testing (Sandoval 2005). Also, if a single body part is tested more than once, it is important that residue of previously tested solutions is removed (Hock et al. 2007), e.g., by rinsing sensilla and then blotting them dry between tests and letting the sensillum return to a normal resting state (Zhou et al. 2021). When sensilla are tested by removing the body part they are on, e.g., to avoid disruption from muscle activity, responsiveness of the sensilla may reduce with time since amputation (van der Molen et al. 1985). When behavioral tests are repeated on the same individual, the insect should be kept from feeding, to avoid changes in response that might result from decreased hunger. When retesting the same individual, the possibility of sensitization or habituation, including cross-habituation, also has to be taken into account (Paranjpe et al. 2012, Ali et al. 2021). Sensitization is an increase in response with repeated exposure. Habituation is a reduction in response with repeated exposure. Cross-habituation is when habituation generalizes from one tastant to other tastant(s), e.g., when exposure to one bitter tastant reduces response to other bitter tastant(s) (Zhou et al. 2021).

Habituation or cross habituation can be avoided by testing each individual or sensillum or neuron only once. When this is not feasible, then the order of presentation can be randomized (e.g., Sandoval 2005, Despland et al. 2011, Carle et al. 2015, Biolchini et al. 2017) or alternated (e.g., Rogers and Newland 2000, de Brito Sanchez et al. 2005, Merivee et al. 2007). Another common solution for electrophysiological tests is to test a single sensillum (or group of sensilla or neurons) from low to high concentration of tastants, but with a few minutes between stimuli to allow disadaptation (e.g., Mitchell and Gregory 1979, Jiang et al. 2015). The rationale is that if there is still any effect of repeated exposure, then the effect is likely to be a decrease in response, whereas increased concentration is expected to result in an increase in response. Thus, if an increased response is seen, a response to concentration, not habituation, is assumed.

### Electrophysiological Evidence

Electrophysiological recordings are of action potential activity of sense neuron(s) upon presentation with a tastant, in comparison to presentation with a control. The recording may be of a particular neuron in a sensillum, of an individual sensillum, or of groups of sensilla, e.g., an entire larval sense organ, such as the terminal organ (TO) of a fly (Shields and Martin 2012). When a recording is from an entire sensillum, it may still be possible to separately identify the action potentials coming from different neurons, or at least some of the different neurons. This is because the firing spikes (= action potentials = impulses) of different neurons differ in shape, amplitude, and temporal pattern (e.g., Fig. 1 of Bernays et al. 2000, Seada et al. 2016). A more reliable way to document activity of an individual neuron is by calcium imaging, i.e., detecting the influx of Ca^2+^ ions that accompanies the electrical activity of a neuron (Apostolopoulou et al. 2016, Raad et al. 2016, Maier et al. 2021).

The possibility that a response is strictly mechanosensory, e.g., is a response to contact with the tastant rather than a response to the particular chemical features of that specific compound, should be ruled out. Popescu et al. (2013) suggest that ‘strong lateral movements’ of a sensillum, not just contact with an electrode, are necessary to generate a detectable response. Chun and Schoonhoven (1973) report that ‘even small deflections’ of certain taste sensilla generate a response, but such responses are detectable because they are smaller than responses of chemosensory neurons in sensilla. Care should be taken to avoid contacting the mechanosensory neuron. Some studies examining taste do not analyze the very beginning of recordings, although the amount of time they skip at the start of the recording after initial contact has varied: e.g., 10 ms (Biolchini 2016, Loy et al. 2016), 50 ms (Blom 1978), 150 ms (Inoue et al. 2009), 200–700 ms (Ling et al. 2014). One reason for skipping the start of the recording is to avoid any response resulting from contact (Biolchini 2016); another reason is to allow the electrical impulse pattern to stabilize (Dethier and Crnjar 1982, Inoue et al. 2009). Some studies conclude they have found evidence of gustation, e.g., by a particular body part, based simply on observing different responses to different nonvolatile solutions. Such studies are implicitly assuming that there are no viscosity differences among the test solutions, or that any differences are too small to cause a difference in mechanosensory response.

When collecting electrophysiological data, damage to the neurons caused by the tastant compound should be ruled out (Bestea et al. 2021). Also, the technique requires the use of a recording electrolyte, e.g., KCl, to help conduct the electrical response, and it is important to ensure that the response is not unduly influenced by the recording electrolyte (van Loon et al. 2008). In honey bees, some taste neurons respond to even the low KCl concentration used as a recording electrolyte (discussed in Bestea et al. 2021), but use of a different recording electrolyte can avoid this issue (Delventhal et al. 2014).

### Behavioral Evidence

Behavioral responses to tastants provide different information than electrophysiological responses because taste responses involve neural processing beyond the level of taste sensilla. Thus, the two types of responses may not always be positively correlated across different tastants or concentrations of a tastant.

A common type of behavioral evidence of taste is PER (described above). To test the effectiveness of bitter compounds, the compound is typically mixed into a sugar solution to see if the percent responding with PER decreases. Some insects also respond to certain tastants by spreading their labellum (Zhou et al. 2019), ceasing walking (Mann et al. 2013, Thoma et al. 2017), or lifting their legs, as grasshopper nymphs do in response to nicotine hydrogen tartrate (White and Chapman 1990). As noted previously, insects use taste to assess other substrates besides just food, e.g., to assess oviposition sites, mates, or need to groom, in which case the behavior to be measured would change accordingly (Boullis et al. 2021). Insects are often given water before testing so that a need for the water in the solutions does not reduce the animal’s differentiation among solutions (e.g., Blystone 2015).

Sometimes whether a body part or sensillum has a taste function is determined by seeing whether the behavioral response to tastants disappears when that body part or sensillum is disabled or removed, e.g., using surgery (Yosano et al. 2020), glue (Becker and Peters 1989), or alteration of genes (e.g., Apostolopoulou et al. 2016). In *D. melanogaster*, *Poxn* mutants have external chemosensory bristles that are replaced by mechanosensory bristles, but the internal pharyngeal taste neurons are intact (Nottebohm et al. 1994, Chen et al. 2018). Thus, if a wildtype, but not the mutant, responds to a tastant, this suggests that the wildtype’s response is through external (peripheral) chemosensilla, not via pharyngeal chemosensilla. Alternatively, evidence of taste by internal body parts can be established if the taste response is seen even after interfering with the ability of external body parts to taste (Miles 1958).

Certain sex pheromones are detected by taste. They are called contact pheromones because response to them occurs only after contact, not from a distance (Blomquist and Ginzel 2021). If a compound is known to be nonvolatile, this rules out olfaction. However, as noted previously, a compound having some volatility does not rule out taste because such compounds may be both smelled and tasted (reviewed in Montell 2021).

### Morphological and Neuroanatomical Evidence

Knowing what body part a sensillum is on is not sufficient to determine its function. For example, antennal sensilla can be gustatory (King and Gunathunga 2023), olfactory (e.g., Li et al. 2018), thermosensory (e.g., Schneider et al. 2018), hygrosensory, or strictly mechanosensory (respond to movement of the sensillum) (e.g., Wang et al. 2018a). The shape of a sensillum alone, e.g., whether it is chaetica, basiconica, trichodea, is also not a reliable indicator of whether a sensillum’s function is gustatory (Mitchell et al. 1999, Table 1 in Barsics et al. 2014). This has often been noted (e.g., Altner et al. 1981, Acevedo et al. 2009, Düngelhoef and Schmitt 2010), yet is still sometimes unrecognized. For example, Yuvaraj et al. (2018) suggest that sensilla chaetica are gustatory. Although some sensilla chaetica appear to be gustatory (e.g., Jung 2015, Ruschioni et al. 2015, Rebora et al. 2016b), others do not (Zacharuk and Shields 1991, Shanbhag et al. 1999, Maher and Thiery 2004). There are, however, specific morphological and neuroanatomical features of sensilla that are useful for assessing whether or not a particular sensillum is likely gustatory (Table 1).

**Table 1.** Morphology and neuroanatomy useful in distinguishing gustatory sensilla from sensilla with alternative functions

Function	Features	Reference
Gustatory sensilla	Single apical or subapical (terminal) pore^a^ (for tastant entry) and: • Each neuron has only one dendrite • Minimal or no branching of dendrites • No lamellated dendrites • Few or no pores on sensillum wall • Lymph (not just dendrites) visible at tip of sensillum •vIn body location able to contact tastants	Altner et al. 1978, Altner and Loftus 1985, Bogner et al. 1986, Zhao et al. 2013, Ban et al. 2015, Schneider et al. 2018
Gustatory and mechanosensory sensilla	• As above. • Also, one neuron^b^ has a tubular body^c^, and at least one neuron does not	Keil 1997
Mechanosensory sensilla	• Aporous or occasionally a terminal pore • A single dendrite with a terminal body	Devitt and Smith 1982, Keil 1997
Hygro/ Thermal sensilla	• Typically aporous, but some appear uniporous • Tend to be protected from wind by surrounding cuticular structures • Lamellations distally on dendrites	Altner and Prillinger 1980, Craig and Batz 1982, Altner et al. 1983, Steinbrecht 1984, Figs. 2 and 4 in Altner and Loftus 1985, Sen 1988, Fig. 1A, B in Tichy and Kallina 2010, Fig. 1 in Schneider et al. 2018, Rebora et al. 2019
Olfactory sensilla	• Multiple wall pores • Pores perhaps smaller in size compared to pores in uniporous sensilla • Dendrites often but not always branched	Stocker 1994, Ochieng et al. 2000, Shields and Hildebrand 2001, Diehl et al. 2003, Schoonhoven et al. 2005, Sevarika et al. 2021
Molting pore	• Single pore but not always apical or subapical, e.g., sometimes basal • Pore present in later instar stages but not in first instar	Wensler and Filshie 1969, Zacharuk et al. 1977, Zacharuk 1980, Honda and Ishikawa 1987

^a^Often difficult to distinguish from pore with strictly molting function, as discussed in text.

^b^Two neurons each with tubular body in an ant species (Dumpert 1972).

^c^For clear cross section illustrating a gustatory neuron with tubular body, see Fig. 2H of Gal et al. (2014), Fig. 4d of Sevarika et al. (2021).

In practice, a single apical pore (uniporous) seems to be the ultrastructural feature of sensilla that is most often used to suggest a gustatory function (e.g., Zacharuk and Shields 1991, He et al. 2019, Taszakowski et al. 2019). Typically, the pore is apical (terminal) or subapical. One difficulty with relying on just the presence or absence of such a pore is that the pore may be hard to see, e.g., because of the drying process in preparation for electron microscopy (Valencia and Rice 1982, Wang et al. 2018b) or because it is covered by digits on the sensillum (e.g., Fig. 39 in Faucheux 2013), or by exudate released by sensilla. The groove-pegged sensilla on the antennae of *Ae. aegypti* were initially described as uniporous (Davis 1988). However, the terminal pore is not consistently seen and may be an artifact of imaging (Cribb and Jones 1995).

Another difficulty with relying on uniporous to assign function is that a pore used for taste can be difficult to distinguish from a pore that is strictly molting yet is at or near the tip of the sensillum (Zacharuk et al. 1977, Figs. 6–8, 10 in Eilers et al. 2012, Fig. 7 in Faucheux 2012, Fig. 4 in Xu et al. 2017a). The dendritic sheath(s) that surround dendrites in sensilla appear to molt away through the molting pore (also called ecdysial pore, basal pore, molting scar, and ecdysial scar) (Wensler and Filshie 1969, Zacharuk 1980). Pores that have been described as molting include basal ones, but also apical ones. When looking at SEM (Scanning Electron Micrographs) of sensilla with a pore at or near the tip, before looking at the figure’s legend, it is difficult to predict whether the pore will be described as a molting pore or as the terminal pore of a taste sensillum. If a pore is present in later instars but not in the first instar, this suggests it may be a molting pore, because first instars would not yet have molted (Honda and Ishikawa 1987); however, sensilla images for first instars along with later instars are rare. Specific internal features of molting pores that can be seen in TEM (Transmission Electron Micrographs) but not in SEM have been proposed with the suggestion that these features are what allow a pore to be used in molting, rather than in letting tastants into the sensillum as a terminal pore does (Chu-Wang and Axtell 1972a). However, how the features logically would facilitate molting is unclear, and whether these features reliably reveal whether a pore is molting needs further analysis. Some molting pores are described as plugged (Zacharuk et al. 1977, Zwicker et al. 2004, Rebora et al. 2007, Tichy and Kallina 2010). However, not all pores that have been labeled as molting pores appear plugged (Fig. 4a in Xu et al. 2017a), and the terminal pore of taste sensilla also sometimes appears plugged (e.g., Rist and Thum 2017). Perhaps, in gustatory sensilla, a terminal pore may be used both for molting and for tastants to enter (Rist and Thum 2017), at least at times when the pore is not plugged with an exuvium. A formal analysis is needed to determine if molting pores are consistently a different size compared to the uniporous pores that are used to conclude a sensillum has taste function. When a pore is deemed molting, the features that led to that conclusion should be explicit; they often are not.

A terminal pore has been found in some sensilla that otherwise appear not to be gustatory. Such is the case for some sensilla that appear to be mechanosensory and not gustatory, based on other features (Chu-Wang and Axtell 1972b, Zacharuk et al. 1977, Fig. 5g in Singh and Singh 1984, Rist and Thum 2017); yet mechanosensory sensilla are expected to be aporous (Devitt and Smith 1982). Some hygro and/or thermal sensilla appear uniporous (Altner et al. 1983, Steinbrecht 1984, Sen 1988), but hygro and/or thermal sensilla are typically aporous, or have multiple wall pores when they are also olfactory (Shields and Martin 2012). That the observed terminal pore is strictly a molting pore in all of these cases has not been ruled out.

Location of a sensillum can be helpful in distinguishing taste sensilla from thermo- and hygro- sensilla, or at least in determining that assigning function should await additional evidence. Gustatory sensilla need to be able to contact their tastants, whereas thermo- and hygro sensilla tend to be protected from wind by cuticular structures around them (Fig. 1A, B in Tichy and Kallina 2010, Fig. 1 in Schneider et al. 2018, Rebora et al. 2019). Being only on body surfaces that routinely contact substrates has been used to suggest a gustatory function, e.g., being only on the ventral apical surface of the antennae (Ruschioni et al. 2012). Formal evaluation of the proportion of sensilla of each function that is so located remains to be done.

Although being multiporous is typically taken as evidence that a sensillum is olfactory (Table 1), this is only a hypothesis. Exceptions, i.e., multiporous gustatory sensilla, have been proposed (Table 2). Of these, the CHC (cuticular hydrocarbons) sensilla of ants provide the best support, but in their case, perhaps it is just a matter of semantics whether they are described as having extended range gustation or limited range olfaction. Among the parasitoid wasps that have been suggested to have multiporous gustatory sensilla, either electrophysiological data are available but stronger evidence of multi-porosity is needed, or multi-porosity is clear, but electrophysiological or behavioral evidence of response to nonvolatile compounds is needed.

**Table 2.** Examples of multiporous sensilla with some gustatory features

Type &/or body location	Organism	Gustatory features^a^	Olfactory features^a^	Other features	References
Multiporous CHC sensilla (antennae)	carpenter ants (Hymenoptera: Formicidae)	• electrophysiological response to long-chain CHCs thought to be non,- or barely,-volatile	• many tiny pores on sensillum wall • ORN-specific receptor proteins • appear to project to glomeruli in ALs • response upon contact or ~1 cm	• >130 neurons • response to CHC blend from nonnest mates	Ozaki et al. 2005, Brandstaetter et al. 2008, Nakanishi et al. 2009, 2010, Ozaki and Wada-Katsumata 2010
Multi-apical-porous sensilla (antennae)^b^	parasitoid wasps *Amitus spiniferus* (Brèthes), *Telenomus busseolae* Gahan, *Trissolcus basalis* (Wollaston) (Hymenoptera: Platygastridae)	• sensilla on apical and/or ventral surface of antennae, which would contact substrates	• multiple pores	• 100s of neurons • pores apical, not on wall	Isidoro et al. 2001, Zhang et al. 2015a
Multi-ventral-porous sensilla (antennae)^b^	parasitoid wasps *Trichogramma* species (Hymenoptera: Trichogrammatidae)	• pores only on ventral margin of sensilla, which would contact substrates • sensilla on apical ventral surface of antennae	• multiple pores	• 10 sensory neurons	Ruschioni et al. 2012 and references therein
‘Apparently ultiparous’ sensilla (antennae)	parasitoid wasp *Diadromus pulchellus* (Wesmael) (Hymenoptera: Ichneumonidae)	• electrophysiological response to protein extract from their hosts’ cocoons, and from which small molecules had been removed; reported to be nonvolatile	• multiple pores are suggested, but clearer evidence is needed	apparent pores apical, not on wall	Bénédet et al. 2002
‘Uniporous- multiporous sensilla’ (proboscis external surface)	Lepidoptera	•a n apical pore • tubular body, i.e., mechanosensory neuron, accompanying the chemosensory neurons • electrophysiological response to sugars and amino acids	• multiple pores in grooves on sensillum wall		reviewed in Faucheux 2013, Guo et al. 2018
Uniporous- multiporous sensilla (antennae ventral surface)	sawfly *Acantholyda posticalis* Matsumura (Hymenoptera: Pamphiliidae)	•a subapical pore	• many small wall pores • branched dendrites	• sensilla concentrated on most proximal of 20–23 flagellomeres	Yuan et al. 2013

^a^Features that suggest, or that original authors use to suggest, this function.

^b^Have been called multiporous gustatory sensilla (MGS) (Isidoro et al. 1996), but gustatory function is just a hypothesis. Have also been called falcate sensilla, sole chercheuse, and multiporous pitted sensilla trichoid C (references in Ruschioni et al. 2012). In the absence of other data suggesting gustatory function of a sensillum, we consider ventral apical location on antennae to be weak evidence for gustation because sensilla with other functions are also frequently ventral and apical.

Another type of sensilla for which additional data on function are needed is the uniporous-multiporous sensilla in some insects (Table 2). These have a terminal pore (a ‘pore tip’) but also multiple wall pores. They are best known in Lepidoptera, but have also been found in a sawfly (Hymenoptera).

For sensilla that have relatively few pores or slits, not many, and only apically (e.g., Zacharuk and Blue 1971, Fig. 3B in Yuan et al. 2013), again electrophysiological and behavioral data would be helpful to determine function. Such sensilla have been suggested to be gustatory (Zacharuk and Blue 1971) or olfactory (Yuan et al. 2013).

Multiporous sensilla, which are typically assumed to be olfactory, seem generally to have smaller pores than uniporous sensilla (Schoonhoven et al. 2005). However, formal analysis is needed. A preliminary examination of some references with data on pore diameter reveals considerable overlap, with multiporous sensilla with pores of 6–70 nm diameter or slits 10–400 nm long (Behan and Ryan 1978, Steinbrecht 1997, Faucheux 2013), and uniporous sensilla with pores 10–400 nm in diameter (Wensler and Filshie 1969, Bassemir and Hansen 1980, Devitt and Smith 1982, Kapoor 1989, Romani et al. 2005, Schoonhoven et al. 2005, Giglio et al. 2009, Shields 2009, Faucheux 2013, Yuan et al. 2013, Rebora et al. 2014, Ban et al. 2015, Zhang et al. 2015b).

In a taste sensillum, each neuron has only one dendrite, and that dendrite is typically unbranched; however, branching has been reported in a few putative taste sensilla (Fig. 14 in Devitt and Smith 1982, Jensen and Zacharuk 1991, Figs. 8–9 in Crook et al. 2008, Giglio et al. 2009). In most taste sensilla, the dendrites are much less branched than in olfactory sensilla that have branched dendrites (Devitt and Smith 1982, Shields 1994, Crook et al. 2008). In spongy moth caterpillars, *Lymantria dispar* (L.) (Lepidoptera: Erebidae), terminal branching of one or two dendrite tips occurs in 1% of sensilla that appear to be gustatory based on electrophysiological responses and other aspects of structure (Shields 2009). TEM are helpful for determining function because they can reveal small features and internal features that cannot be seen with SEM, e.g., dendrites and their features. If TEM through multiple levels of a sensillum show more dendrites near the tip than proximally, this indicates branching, which is typically indicative of olfaction (Table 1). Contrary to Zhou et al. (2008), the reverse pattern does not suggest branching, but rather indicates that not all dendrites reach the tip.

There appears to be consensus that a sensillum has gustatory function if it has a neuron with a tubular body distally, i.e., a mechanosensory neuron, provided it also has at least one other neuron lacking a tubular body (Amrein 2016). Electrophysiological recordings show gustatory and mechanosensory function in sensilla with such ultrastructure (Wolbarsht and Dethier 1958, Isidoro et al. 1998). Even when a pore has not been documented, such sensilla have been assumed to be gustatory (Wang et al. 2018a, Sevarika et al. 2020). The mechanosensory neuron stops at the cuticular base of the sensillum (Fig. 14 in Faucheux 2013, Rist and Thum 2017). However, not all neurons that terminate proximally, instead of extending to the sensillum’s tip, have a tubular body (e.g., Valencia and Rice 1982).

Having some sensilla with both gustatory and mechanosensory functions as inferred by the ultrastructure of the sensillum is extremely common. However, the assertion that all taste sensilla have a mechanosensory neuron and function (e.g., Shanbhag et al. 2001, Amrein 2016) is not correct. Some taste sensilla, even some external taste sensilla, lack a tubular body, i.e., a mechanosensory neuron, and instead are unisensory, having just a gustatory function (Pappas and Larsen 1976, Becker and Peters 1989, Fig. 10 in Rist and Thum 2017). Sensilla that have been shown electrophysiologically to have a taste response but not a mechanosensory response (Rice et al. 1973, Merivee et al. 2004) presumably lack a tubular body, although this still needs to be confirmed. Sensilla that are both gustatory and mechanosensory have a flexible socket, like sensilla that are strictly mechanosensory; whereas strictly gustatory sensilla that are not also mechanosensory have an inflexible socket (Altner and Prillinger 1980, Romani et al. 2005, Ruschioni et al. 2015, Wang et al. 2018a).

Where a sensillum’s axons project to in the CNS used to be considered very reliable for distinguishing gustatory sensilla from olfactory ones (Kwon et al. 2006). The axons from olfactory sensilla on the antennae and maxillary palps typically project to glomeruli within the antennal lobes (ALs) of the brain (Laissue and Vosshall 2008, Lin et al. 2018, Maeda et al. 2020). Hence the ALs have been called the primary olfactory center (Gao et al. 2000, Rybak et al. 2016). The subesophageal ganglion or zone (SOG or SEZ or gnathal ganglion) has been called the primary gustatory center (e.g., Maeda et al. 2014, Sun et al. 2021b); and consistent with this, axons from many mouthpart gustatory sensilla project to the SOG/SEZ or SOG/tritocerebrum, e.g., in various fly larvae and caterpillars (Kvello et al. 2010, Tang et al. 2014, Kendroud et al. 2018, and references therein). However, some axons of some gustatory sensilla also project to other parts of the CNS. For example, in the kissing bug *Rhodnius prolixus* Stål (Hemiptera: Reduviidae), axons of antennal gustatory sensilla project to the antennal lobes (Pontes et al. 2022). In the blowfly *Ph. regina*, axons of the gustatory sensilla in the tarsi project to thoracic ganglia and SOG (Edgecomb and Murdock 1992). In the migratory locust *Locusta migratoria* L. (Orthoptera: Acrididae), axons of the gustatory sensilla on the genital organs project to abdominal ganglia (Tousson and Hustert 2000). In addition, some olfactory receptor neurons on the maxilla of the blow fly, *Ph. regina*, project to the SOG, which allows them to interact with projections from labellar GRNs (Maeda et al. 2014).

## Summary of Suggestions for Future Studies

Some suggestions for future studies were brought up in this review and include the following: Further research is needed on what aspects of a sensillum’s morphology and neuroanatomy reliably reveal function. In a review of insect sensilla, Zacharuk (1985) called for more studies that combine ultrastructural data, e.g., from TEM, with electrophysiological data, in hopes of being able to assign function from morphology with greater confidence. This is still needed. We additionally note that a formal analysis of whether there is a size difference between molting pores and uniporous pores might be useful, because criteria for distinguishing these two types of pores is not yet clear. Such distinction is important because ‘uniporous’, but not a single molting pore, is frequently used to conclude that a sensillum has taste function. Additional exploration of the function of multiporous sensilla that are suspected to be gustatory (Table 2) also is needed.

Future studies of insect taste should always provide explicit information on: i) the criteria that was used to distinguish between a molting pore and a pore that can be used to conclude taste function; ii) the basis for assigning a tastant to one of the taste qualities, e.g., sweet: is it based on human taste perception, on whether the insect’s response is deterrent or appetitive, or on chemical structure? iii) the basis for concluding a taste function, e.g., as opposed to an olfactory or strictly mechanosensory function, e.g., is it based on the compound being nonvolatile, based on specific sensilla features, and/or based on evidence from a similarly located sensillum in a closely related species?

Some taste terms can be confusing, so clarity for a broad readership needs to be considered. In the past, usage has been inconsistent for the terms unimodal versus unisensory and multimodal versus multisensory. We suggest that modes are within senses, and thus ‘modal’ be used to refer to within a taste quality and ‘sensory’ be used to refer to the different senses (gustation, olfaction, mechanosensation, etc.). ‘Sugar best cell” is typically preferable to ‘‘sugar cell’. GR is an abbreviation for ‘gustatory receptor’; so if a receptor is known to be in the GR clade but its function is unknown, this should be made clear. Likewise, there are clade names within GR, such as bitter GR. Whether ‘bitter GR’ is being used to indicate that the function is known or that the clade is known should be made clear. For the concept of taste in insects to be narrow enough to be useful and for closer consistency with how the term taste is used by those in other fields of study, and by non-scientists, we suggest that responses be deemed taste only when a sensillum is involved.
